# Research on APT groups malware classification based on TCN-GAN

**DOI:** 10.1371/journal.pone.0323377

**Published:** 2025-06-10

**Authors:** Daowei Chen, Hongsheng Yan

**Affiliations:** School of Information and Communication, National University of Defense Technology, Wuhan, China; Air Force Engineering University, CHINA

## Abstract

Advanced Persistent Threat (APT) malware attacks, characterized by their stealth, persistence, and high destructiveness, have become a critical focus in cybersecurity defense for large organizations. Verifying and identifying the sources and affiliated groups of APT malware is one of the effective means to counter APT attacks. This paper addresses the issue of tracing and attributing APT malware groups. By improving and innovating the extraction methods for image features and disassembled instruction N-gram features of APT malware, and based on the Temporal Convolutional Network (TCN) model, the paper achieves high-accuracy classification and identification of APT malware. To mitigate the impact of insufficient APT malware samples and data imbalance on classification performance, the paper employs Generative Adversarial Networks (GAN) to expand the sample size. Validation on both public and self-constructed datasets shows that the proposed method achieves an accuracy and precision rate of 99.8%, significantly outperforming other methods. This work provides a foundation for subsequent countermeasures and accountability against related APT attack groups.

## 1 Introduction

APT (Advanced Persistent Threat) attacks refer to targeted cyberattacks aimed at specific organizations and objectives. Unlike conventional cyberattacks, APT attacks are characterized by their advanced methods, prolonged duration, and highly specific targets. These attacks can be launched against industries, power systems, financial systems, [1,2] and typically carried out by sophisticated hacker groups or state-sponsored groups, making them particularly challenging to defend against and identify. In recent years, the variants of APT malware have been evolving at an accelerated pace, and the groups behind these attacks have become increasingly diverse and difficult to trace. According to the “2024 Mid-Year Global APT Attack Activity Report” [3], APT attacks by major nations remained highly active and frequent in 2024. Among the most active APT groups were North Korea’s Kimsuky and APT44, APT28, and Pakistan’s Transparent Tribe, among others. Given the growing proliferation of APT malware variants and their associated groups, the rapid traceability and attribution of these threats have become a critical issue worthy of in-depth research.

Currently, traditional static analysis and classification methods based on known features have achieved relatively good results in classifying conventional malware. However, these methods exhibit certain limitations when dealing with unknown malware. In recent years, an increasing number of scholars have begun to employ artificial intelligence (AI) techniques, Including the use of machine learning or deep learning to detect and identify malicious attacks [4]. AI-based detection methods can circumvent the complexity and inefficiency of manual detection, streamline the analysis process, and enhance classification performance.

Malware classification methods based on artificial intelligence technologies have achieved significant research progress. Based on the types of features extracted, these methods can be divided into single-feature and multi-feature classification approaches. For example, Priya V *et al*. [5] investigated single-feature malware image characteristics and proposed a novel malware image representation method based on the Gray-Level Co-occurrence Matrix (GLCM). The aim was to maintain the stability of image feature dimensions, avoid resizing image features, and thereby accelerate the training of CNN classifiers. Zhang D *et al*. [6] introduced the MalMKNet model based on grayscale images for malware classification and identification. The core idea was to improve the CNN model through multi-scale kernel fusion, and experimental validation on the Malimg dataset demonstrated a significant improvement in detection performance. Wu P *et al*. [7] utilized deep learning algorithms to fuse API presence features and API transition features based on API call sequences of malware, proposing the MINES method, which achieved excellent results across five datasets. Zhang D *et al*. [8] proposed a multi-scale kernel malware classification method (IMCMK) based on image features, employing a convolutional neural network (CNN) architecture with hybrid multi-scale convolutional kernels to enhance the detection capability of malware variants. Experimental results showed an accuracy rate of up to 99.25%. Qian W *et al*. [9], based on opcode sequence features, introduced the concept of word vectors to extract semantic features of opcodes and then used a text convolutional neural network (textCNN) to achieve malware classification. Experiments demonstrated an accuracy rate of 98% on a publicly available Microsoft dataset.

In terms of multi-feature fusion, Li S *et al*. [10] integrated image features, assembly instruction features, and API features to propose the RGB-MalNet model. This model innovatively transforms malware representations into image channels through RGB three-channel mapping, enhancing information richness and discriminative power. The model achieved accuracy rates of 99.47% and 97.55% on the Kaggle and DataCon datasets, respectively. Xuan B *et al*. [11] utilized the raw features of malware to extract raw binary data, disassembled opcode data, and API data for feature extraction and combination, generating an RGB three-channel pixel fusion map. Subsequently, an improved CNN algorithm was applied to detect the images and classify malware on the Kaggle dataset, achieving a detection accuracy of over 97.78%. Huang W *et al*. [12] addressed the issue of conventional malware classification by leveraging both image features and Opcode features on the Kaggle dataset. Building on the Bidirectional Temporal Convolutional Network (BiTCN), the authors proposed the BiTCN-SA model, which incorporates a self-attention mechanism to improve the accuracy of malware classification. Li S *et al*. [13] further enhanced the BiTCN by introducing a pooling fusion mechanism, proposing the BiTCN-SA model, which achieved a classification accuracy of over 99%.

In the field of APT malware detection and traceability, Du Y *et al*. [14] proposed an organizational classification model based on Graph Attention Networks, which incorporates a multi-head attention mechanism to achieve the classification objective. Liang H *et al*. [15] adopted a 1D-CNN approach based on API features to detect and identify APT malware, achieving a detection probability of 95.8%. However, the rationality of the dataset proportions remains open to further discussion. Jian Z *et al*. [16] focused on the classification of APT malware using a multi-feature hybrid method that combines API features and opcode features, implementing Graph Neural Networks (GNNs) to classify APT malware with an accuracy rate of 94.23%. Shu L *et al*. [17] explored the detection and traceability of APT malware using the SMOTE-RF algorithm, comparing various machine learning algorithms and attaining a classification accuracy exceeding 80%. Gil S *et al*. [18] introduced a temporal learning method named Bon-APT, which analyzes and categorizes the temporal behavior of dynamically called APIs in APT malware and uses machine learning models for detecting and attributing APT attacks. Han W *et al*. [19] presented an APT malware detection and cognition framework called APTMallnsight, which identifies and understands APT malware through system call information and ontological knowledge.Experimental results showed detection and clustering accuracy rates of 99.28% and 98.85%, respectively. Lastly, Bin T *et al*. [20] employed an enhanced Convolutional Neural Network (CNN) model combined with an attention mechanism to identify APT software based on static features, achieving an accuracy rate of 89.8%. Overall, existing methods for APT malware traceability and classification have made significant progress. However, most are limited to specific datasets with small sample sizes and lack scalability.

APT groups malware, characterized by its stealth and sophistication, often propagates and attacks using Trojan viruses, which differs significantly from the behavior of conventional malware families. As a result, traditional malware classification methods may not be suitable for APT malware. To address this challenge, this paper conducts an in-depth study on the traceability and attribution of APT malware. By extracting static feature data independently of traditional sources and leveraging advancements in artificial intelligence technologies, we propose a Temporal Convolutional Network (TCN) model-based approach for APT malware traceability and classification. Compared to traditional machine learning methods, our proposed approach shows significant improvements in both efficiency and accuracy, achieving a high level of classification precision.

## 2 Traceability and classification framework

### 2.1 Model framework design

**Fig 1:**
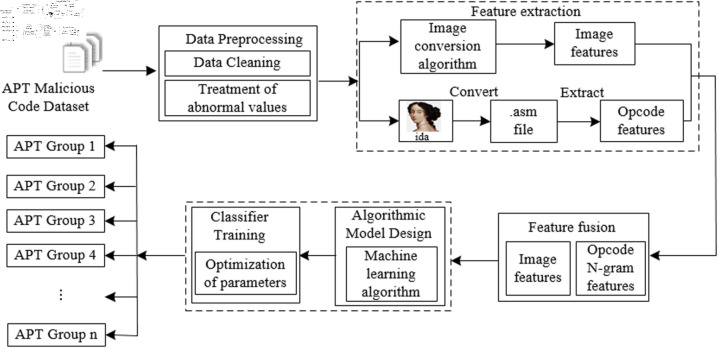
The overall framework for APT malware classification.

To achieve the detection and identification of APT malware, this paper designs a traceability detection process framework as illustrated in Fig 1. This framework encompasses data processing, feature extraction and fusion, and classification training, among other steps, realizing a comprehensive process from the initial raw sample dataset to the final APT malware classification results.

The detailed procedural steps for the implementation of APT malware traceability classification in Fig 1 are as follows:

(1) Constructing the sample dataset: Firstly, it is necessary to build and collect high-quality APT attack sample datasets through publicly available channels. The dataset should contain diverse samples from multiple groups, ensuring representativeness and distribution.

(2) Data preprocessing: The collected APT malware dataset undergoes data preprocessing, including data cleaning and outlier handling. Abnormal samples and those that cannot be converted using IDA are removed, retaining only processable samples to form a high-quality, qualified dataset.

(3) Feature extraction: This step primarily involves extracting image grayscale value features and opcode features. The image grayscale value features are extracted using a binary file image conversion algorithm, while opcode features are obtained by disassembling the software with IDA to extract assembly instruction opcodes. These opcodes are then processed using the N-gram algorithm to form effective opcode features.

(4) Feature fusion: The extracted image features and N-gram opcode features are concatenated and fused, ensuring that the fused data format is suitable for training and processing by different artificial intelligence algorithms.

(5) Machine learning algorithm training: The fused data is fed into the designed machine learning model for training and classification. After model training and parameter optimization, the final prediction results are output.

### 2.2 Dataset selection and preprocessing

**Fig 2:**
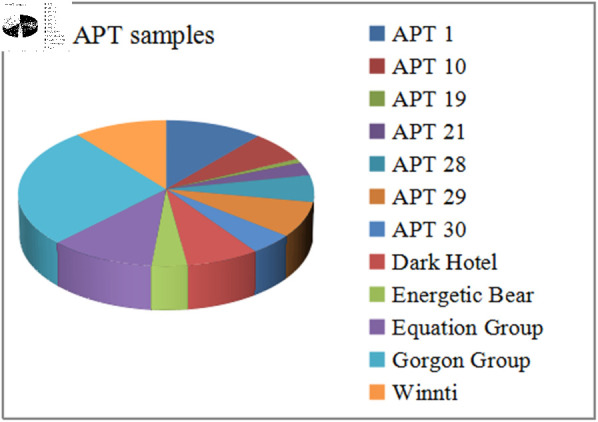
Total number and distribution of APT samples dataset.

To achieve traceability and attribution of APT malware samples, a high-quality and comprehensive dataset is one of the key factors for achieving traceability performance. In existing APT attack cases, due to competitive barriers among security vendors, complete APT attack samples and data are scarce. We obtained samples from major APT attack groups by accessing the GitHub open-source code hosting platform (https://github.com/cyber-research/APTMalware). As shown in the Fig 2, this malware sample dataset consists of a total of 3,594 samples, sourced from 12 APT groups across 5 different countries and regions. The specific number of samples for each APT group is listed in Table 1.

**Table 1:** Public benchmark dataset D1.

Groups Name	Number of original samples	Number of samples processed
APT 1	405	405
APT 10	244	228
APT 19	32	32
APT 21	106	86
APT 28	214	168
APT 29	281	263
APT 30	164	164
Dark Hotel	273	269
Energetic Bear	132	132
Equation Group	395	395
Gorgon Group	961	343
Winnti	387	385
v Total	3594	2870

During the data preprocessing stage, it is necessary to perform preprocessing steps on the acquired APT sample file data, including cleaning and removing useless samples, and unifying formats and data. First, clean samples that cannot be executed, including those that cannot run on the Windows system or samples that lack the necessary DLL files for execution. Secondly, since subsequent feature extraction requires disassembly processing, IDA Pro is used to manually eliminate abnormal samples that cannot be converted into “.asm” files. After the data preprocessing stage, the total number of effectively executable samples is 2870, as shown in Table 1.

Since APT groups and malicious code samples are continuously evolving, new group samples may differ from previous ones. To validate the rationality and scalability of the algorithm model proposed in this paper, relying solely on the aforementioned open-source dataset of 3,594 APT samples presents certain limitations. Therefore, this paper constructs another dataset comprising the latest APT group samples using publicly collected data. This sample dataset is sourced from a public APT attack research website (https://vx-underground.org/), with a total of 274 samples, as detailed in Table 2. Subsequent tests and validations of the proposed models and algorithms will be conducted based on this self-constructed dataset to demonstrate the applicability of the proposed method.

**Table 2:** Self-constructed APT samples dataset D2.

Groups Name	Number of original samples	Number of samples processed	Upload Time
UNC1549	26	22	2024.02.27
Blind Eagle	25	25	2024.03.20
LightSpy	11	11	2024.04.11
APT31	16	16	2024.04.16
DuneQiu	40	40	2024.04.18
APT44	44	25	2024.04.20
Operation Crimson	26	21	2024.06.05
APT41	16	16	2024.07.18
APT45	34	30	2024.07.25
North Korean Threat Groups	36	27	2024.09.09
Total	274	233	

### 2.3 Feature extraction

To extract the key features of APT malicious code for the training and learning of artificial intelligence algorithms, we conducted reverse engineering using the disassembly software IDA Pro 7.6 based on static analysis techniques. Additionally, we developed Python programs to analyze and process APT malicious samples, extracting both opcode features and image Grayscale Value features from the samples.

#### 2.3.1 Image grayscale value feature extraction.

**Fig 3:**
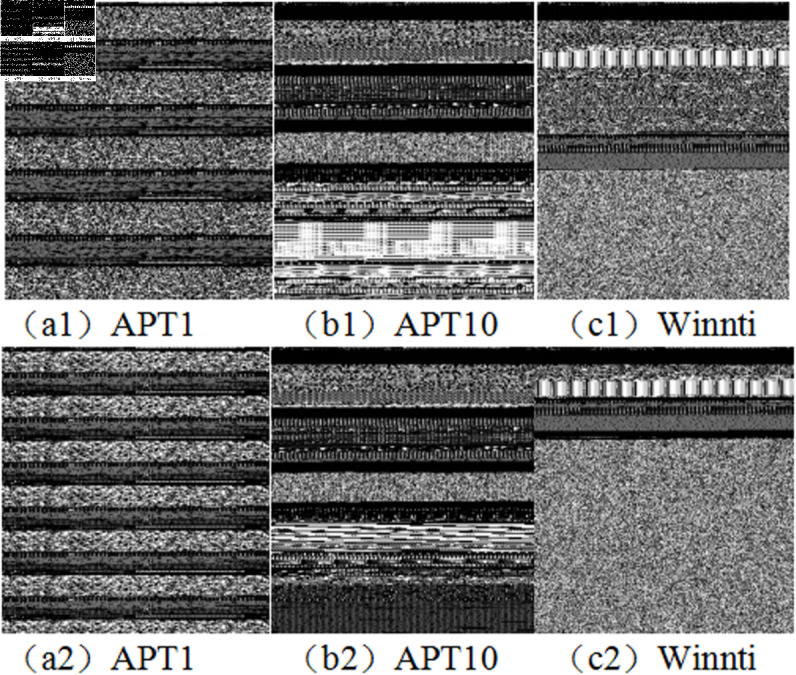
Comparison of APT groups malicious code gray-scale images.

The underlying data of APT malicious code consists of binary bit data. By establishing a corresponding relationship between bytes and grayscale images, the malicious code can be converted into byte data to extract grayscale image features. Malicious code from the same APT group or source, even after variations and reuse, often exhibits similar code structures and data. Consequently, when converted into image features, APT malicious code images tend to display similar texture characteristics. This lays the foundation for tracing and attributing APT malicious code samples. For example, as shown Fig 3 in the grayscale images of three groups of APT group malicious code samples (a1, a2; b1, b2; c1, c2), they exhibit visually identifiable homology.

**Fig 4:**
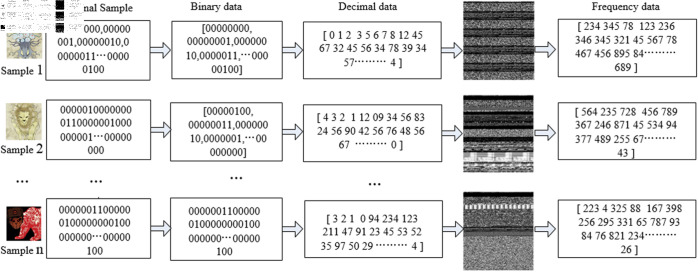
Flowchart of image grayscale value feature acquisition.

Diverging from traditional malicious code detection methods that directly extract image features for classification, this paper aims to achieve classification detection of APT malicious code by altering the extraction of image features to the extraction of image grayscale value features. That is, after converting APT malicious code into images, we further extract the grayscale value features for subsequent algorithmic detection and recognition. The process is illustrated in Fig 4. Initially, the raw samples of the malicious code are obtained and then converted into 8-bit binary data. Subsequently, this binary data is transformed into decimal data within the range of [0-255], thereby acquiring the original grayscale image data values. Following this, the frequency of occurrence of each grayscale value is directly tallied, culminating in the acquisition of feature data consisting of a 256-length grayscale value frequency spectrum. This method of feature extraction diverges from existing literature [21,22] that directly converts malicious code into two-dimensional or three-dimensional image data. Instead, it quantifies the frequency of grayscale values, which not only compresses the data storage volume but also enables a concentrated statistical analysis of similar code segments distributed across different locations within the malicious code, offering superior classification and statistical characteristics.

The aforementioned process can be directly implemented through Python algorithm programming. Specifically, by reading the samples from the APT malicious code dataset using the ’rb’ mode in Python, the sample files can be directly read as binary data streams and stored in byte units. Subsequently, each byte value is converted into a decimal value ranging from 0 to 255. Then, by counting the frequency of occurrence of these decimal values, the grayscale image feature data can be extracted from a single binary sample file. For multiple samples, the complete extraction can be achieved through iterative traversal. The basic design process and execution flow of the algorithm are as follows Algorithm 1:

**Algorithm 1. APT malicious code sample image feature extraction.**



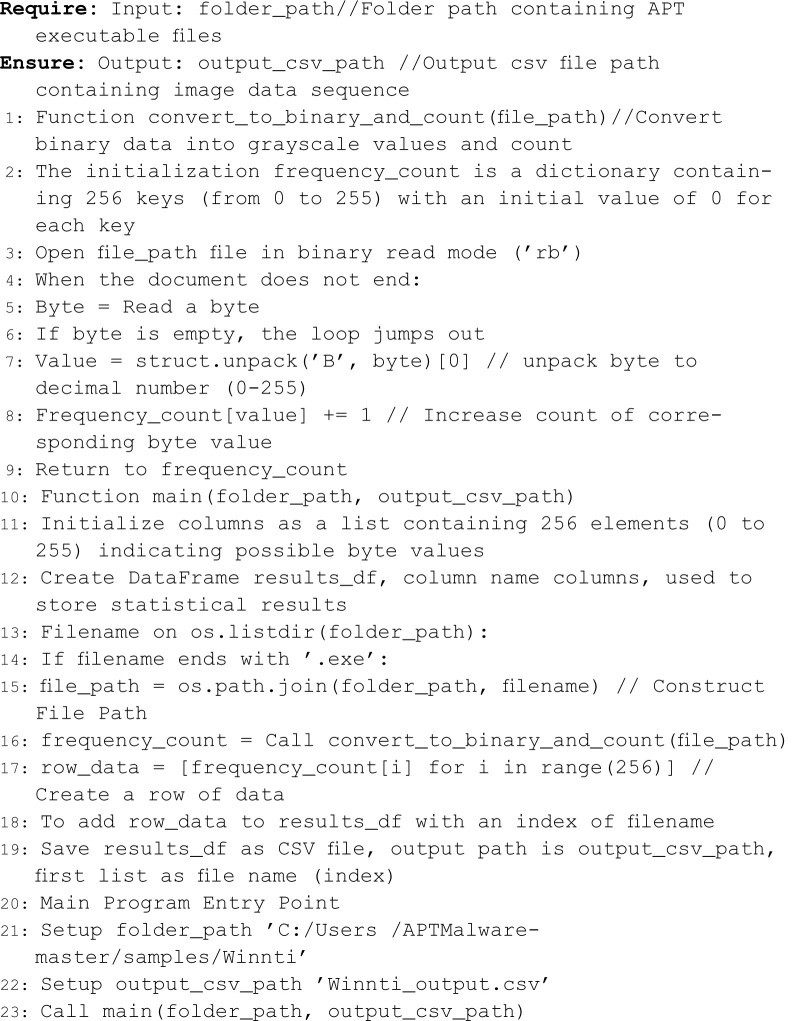



#### 2.3.2 Feature extraction of assembly instruction opcodes.

**Fig 5:**
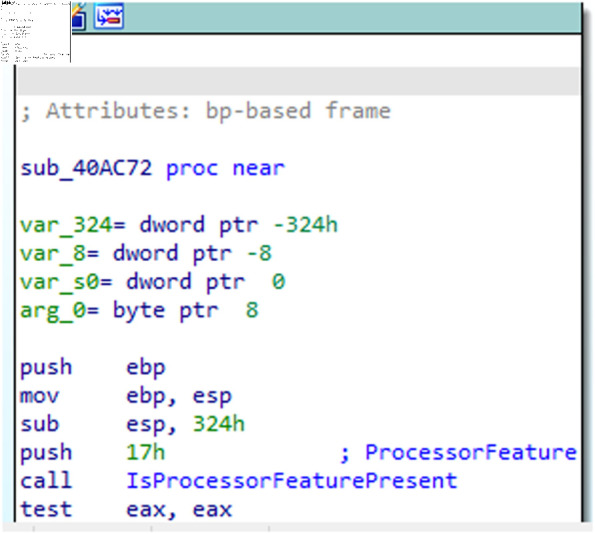
APT malicious code disassembly processing.

APT malware samples are often written in various programming languages. However, regardless of the language used, the malware ultimately needs to be converted into assembly instructions that can be recognized by computer hardware in order to be executed. Therefore, by using disassembly software, the underlying assembly instructions of the malware sample can be obtained, revealing the execution logic and working principles of the entire malware. For example, the result of disassembling a certain APT malware sample is shown in Fig 5. The assembly instructions include operation instructions and address information. For instance, in the statement “push esi,” “push” is the operation instruction, and “esi” is the address information. This statement means placing esi into a register. The operation instructions in assembly instructions are also known as opcodes, which represent the execution actions of each step of the program. Due to code reuse and code variants within APT groups, malware samples from the same APT group often have similar opcodes. This lays the foundation for extracting opcode features to identify the homology of APT malware.

**Fig 6:**
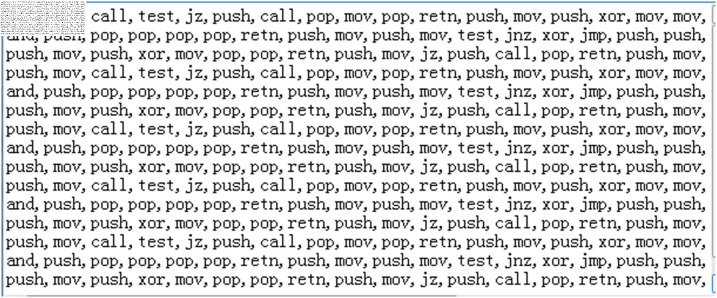
Extraction of APT malicious code opcode instruction.

To extract opcodes from APT group samples, it is necessary to use the reverse engineering tool IDA Pro to convert the samples into .asm files, and then extract the opcodes from them. As shown in the Fig 5, assembly opcode instructions such as “push”, “mov”, and “call” are the ones that need to be extracted. After converting the APT malware sample into an .asm file, the opcodes must be programmatically extracted, and the results are ultimately stored in a txt file. The extracted opcodes are shown in Fig 6.

The extracted opcode instructions, being stored in string format, are not recognizable by subsequent machine learning algorithms. Therefore, it is necessary to utilize the N-gram algorithm [23] to convert them into numerical sequences. The principle behind this is to divide a string into substrings of N characters and to calculate the probability or frequency of their occurrence, with N typically taking values of (1, 2, 3, 4, 5), thereby transforming the string text into a sequence of numbers,The N-gram algorithm is widely applied in the field of Natural Language Processing (NLP). For instance, when N is set to 3, the N-gram algorithm will tally the frequency of occurrence of three consecutive opcode instruction characters such as “push_mov_pop”, thereby extracting the code execution behavior patterns of APT malicious samples and subsequently identifying similar samples. This is because variants of malicious code or samples from the same organization often share similar code fragments and programming logic.

**Fig 7:**
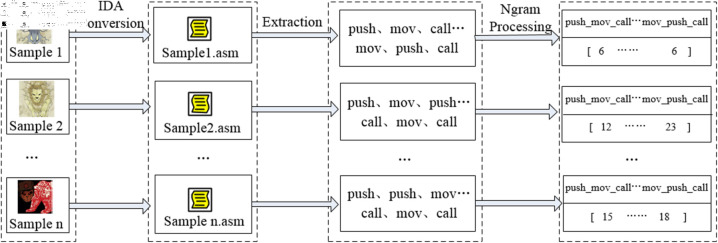
N-gram feature extraction process.

According to the research findings in reference [12], the N-gram algorithm achieves optimal performance when the value of N is set to 3. Consequently, this paper adopts N=3 for extracting the N-gram features of opcode instructions from APT malicious code. The feature extraction process, as illustrated in Fig 7, primarily includes IDA conversion, opcode instruction extraction, and N-gram algorithm processing. Ultimately, the data extracted from all 12 APT organizations are amalgamated to form the opcode instruction feature dataset.

It should be noted that this paper follows the design in reference [13], retaining features with a frequency of more than 500 occurrences for each category. For example, if the N-gram feature “push_mov_add” appears more than 500 times across all samples, it will be retained. This design helps filter out N-gram features with lower occurrence frequencies and preserves those with higher frequencies, thereby capturing the primary operations and behavioral characteristics of APT malware.

#### 2.3.3 Feature concatenation and fusion.

**Fig 8:**
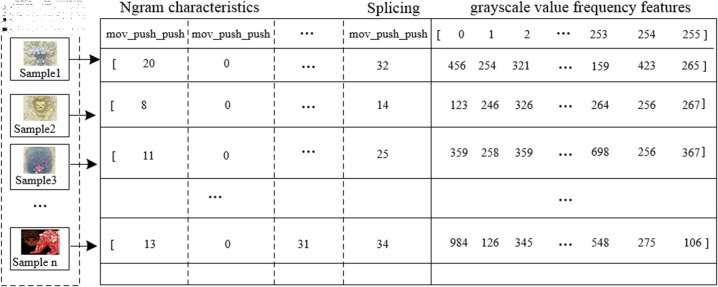
Data concatenation.

After extracting the image grayscale value frequency features and the N-gram features of the opcodes, the next step is to concatenate and fuse the two. The approach adopted in this paper is to directly append the image grayscale value frequency features to the end of the N-gram features. Additionally, a column for APT organization labels is added to the last column of the data for different organizations, resulting in the final dataset. Fig 8 illustrates the schematic of the concatenation of N-gram features with image grayscale value frequency features.

## 3 Detailed design of the TCN-GAN model

### 3.1 Detailed design of the algorithm model

In recent years, convolutional neural network (CNN) models based on image features have made significant research progress in the field of malware detection and classification. By converting malware into image features and leveraging the capabilities of CNNs in image recognition, many algorithmic models have achieved high classification accuracy. For example, reference [11] conducted research on the Kaggle dataset. While CNN models have demonstrated excellent performance in classification tasks, they also have certain limitations and shortcomings:

1. Inability to capture long-range dependencies: CNNs struggle to model dependencies between distant information.
2. High computational resource consumption: CNNs rely on self-attention mechanisms, which can lead to a sharp increase in computational load and memory usage when processing long sequences.
3. Sensitivity to hyperparameters: The performance of CNN models can be highly sensitive to the choice of hyperparameters, such as the number of layers, the number of heads, and the size of hidden layers, requiring careful tuning to achieve optimal performance.

To address these limitations and improve the performance of CNN models, this paper explores various improvements to enhance the efficiency and scalability of the model. After numerous experiments, the final approach adopted is the use of Temporal Convolutional Networks for classification tasks.

**Fig 9:**
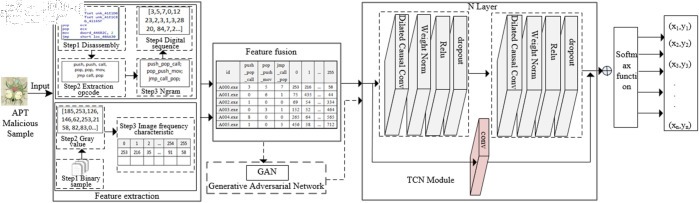
TCN network architecture diagram based on feature fusion.

The TCN is a type of recurrent neural network that excels in processing sequential data and has demonstrated strong performance in classifying and recognizing image features. It can remember long-term dependencies within sequences and is characterized by its simple structure and low computational requirements. Applying the TCN model to APT malware classification and detection not only enables the retention of long-term information within sequences but also allows for flexible focus on key information within the sequences.The TCN based attribution and classification framework using feature fusion is shown in Fig 9. The architecture includes a feature extraction module, a feature fusion module, a GAN-based generative network module for data augmentation in cases of data imbalance, and a TCN neural network training module.

### 3.2 Temporal convolutional network model

In this paper, the TCN neural network training model is adopted for the feature-fused training data. This model was initially proposed and applied in [24]. Compared to other common neural network models, the TCN neural network offers advantages such as a smaller number of parameters, faster training speed, and high classification accuracy for image data. Building on the TCN model, reference [25] introduced a Bidirectional Temporal Convolutional Network.

#### 3.2.1 Basic Structure and design of the TCN model.

**Fig 10:**
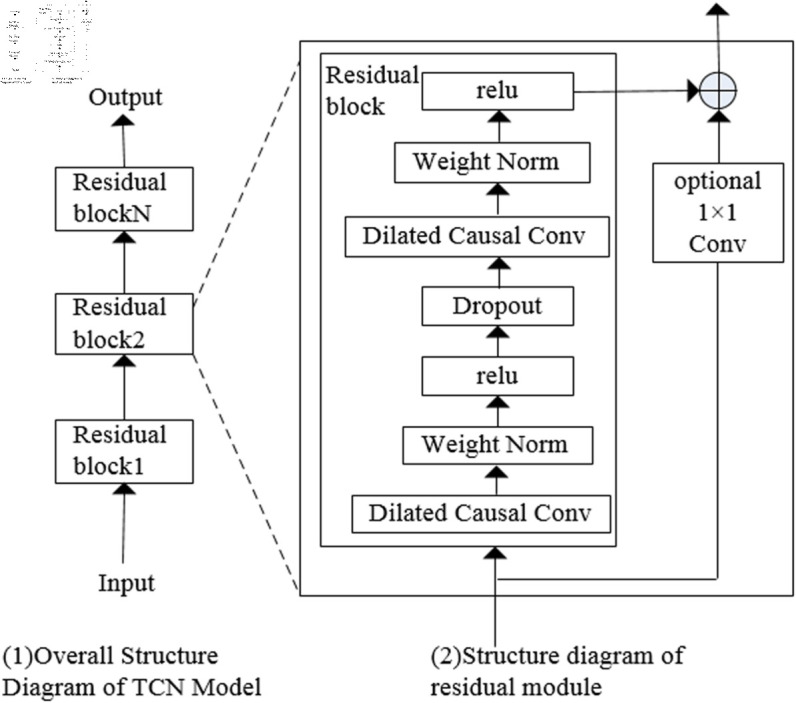
Basic architecture of TCN model.

The main component of the TCN model is the residual connection, which consists of multiple residual connection modules that define the input-output relationship, as illustrated in Fig 10. Each residual module includes two dilated causal convolution operations, normalization, and an activation function.Additionally, a Dropout layer is incorporated to prevent overfitting. The results are then output through the residual connection. Compared to traditional RNNs and CNNs, the TCN model offers significant advantages. These include the ability to leverage filters for parallel processing, thereby accelerating the processing speed of sequences. Furthermore, the TCN model allows for flexible adjustment of the receptive field size. For instance, by stacking convolutional layers, using larger dilation factors, and increasing the number of filters, the model can capture more extensive information sources and distribution patterns.

The progressively increasing receptive field structure of TCN enables it to capture multi-level dependencies between sequences, making it more effective in various time series prediction and classification tasks. The key components of the residual module in the TCN model are the dilated causal convolution, layer normalization to prevent overfitting, and the Dropout layer.

**Fig 11:**
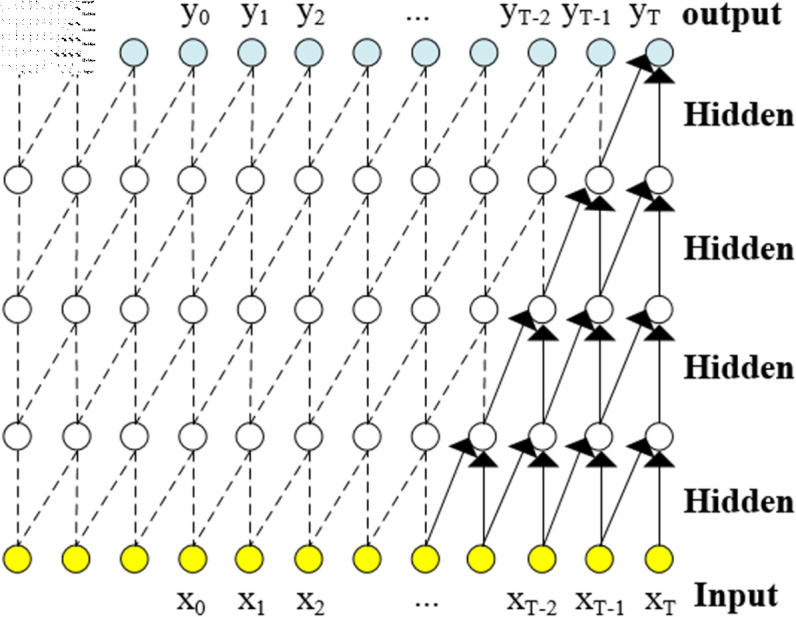
Schematic diagram of causal convolution.

• Dilated Causal Convolution. Dilated causal convolution is a deep convolutional processing model that combines the advantages of dilated convolution and causal convolution. It effectively integrates the strengths of both, enabling the expansion of the receptive field while maintaining causal relationships. Unlike traditional CNN convolution, causal convolution addresses the issue of information leakage, as illustrated in Fig 11.

The calculation formula for causal convolution is as shown in 1. Since causal convolution can only capture historical information that is linearly related to the depth of the network, it faces challenges when applied to sequence tasks, especially those involving sequences with long historical information.

$$y_{T}=F\cdot X=\sum_{k=1}^{K}f_{k}x_{t-K+k}$$ (1)

**Fig 12:**
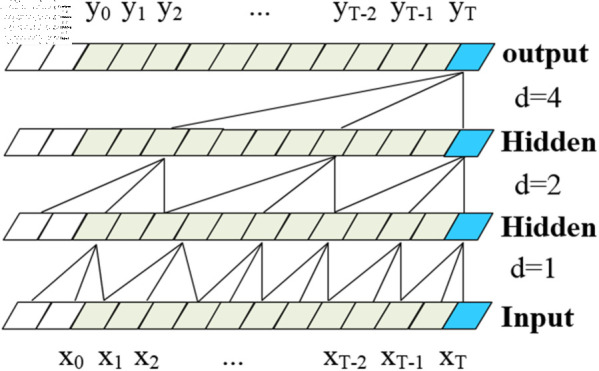
Schematic diagram of expansion convolution.

On the other hand, dilated convolution, also known as atrous convolution or expanded convolution, increases the receptive field exponentially by using a dilation factor, thereby reserving corresponding space. The principle is illustrated in Fig 12. For a one-dimensional input sequence $x\in {\mathbb{R}}^{n}$ And Filters f:{0,1,…,k−1}∈ℝ, The calculation of the dilated convolution for elementin the sequence is shown (2) below:

$$F(s)=\sum_{i=0}^{K-1}f(i)\cdot x_{s-d\cdot i}$$ (2)

Here, represents the dilation factor, denotes the filter size, indicates the position information of the current element, and represents the position information of the input from the previous layer.

• Residual Connection. In the TCN network, to accelerate model stability and convergence, weight normalization and the ReLU activation function are employed. The primary role of the activation function is to further reduce gradients and prevent gradient explosion. The calculation formula for the residual connection is as follows 3:

o=activate(x+f(x)) (3)

After processing through the TCN layers, the model captures the key information and long-range dependencies of APT malware behavior features. These features are then passed through a fully connected layer for linear transformation and a softmax function transformation. Since this is a multi-class classification problem, the softmax function is chosen for multi-class classification. It ultimately converts the output into predicted probability values ranging between 0 and 1.

#### 3.2.2 Bidirectional temporal convolutional network.

**Fig 13:**
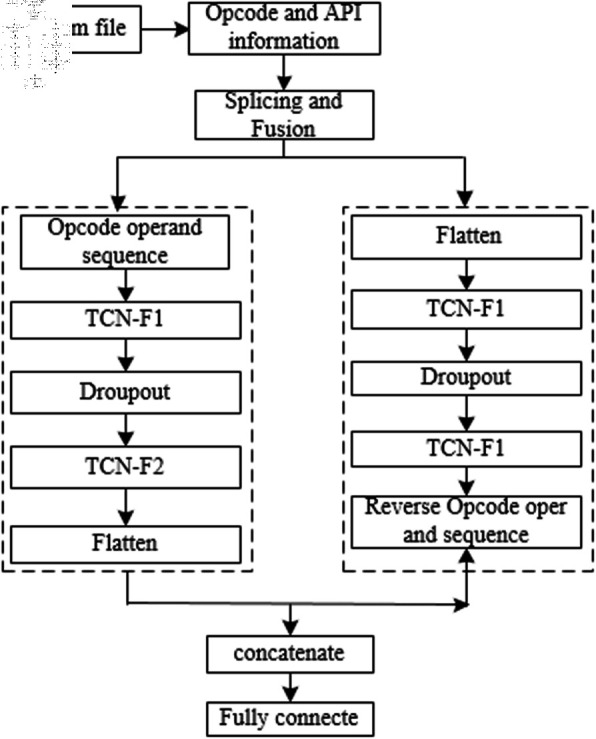
Structure diagram of BITCN network.

The Bidirectional Temporal Convolutional Network (BiTCN) is an extension of the TCN network, combining forward and reverse TCN networks [20]. This approach enables the capture of sequence information from multiple directions, leading to improved performance in certain datasets. As shown in Fig 13, we will also compare its results in subsequent experiments.

### 3.3 CWGAN-GP generative network

For APT attacks, due to their covert and latent nature, the number of malicious code samples collected by defenders is often limited. Moreover, as attack targets and time periods vary, APT groups may develop and deploy different malicious code samples. Therefore, a challenge arises in how to perform feature extraction and training recognition for APT malware with limited samples. In this paper, to address the issue of a limited number of malicious code samples from certain APT organizations, we employ Generative Adversarial Networks to produce a sufficient quantity of high-quality simulated samples. This facilitates the learning process of intelligent models, thereby enhancing the classification and recognition capabilities for new malicious code samples.

**Fig 14:**
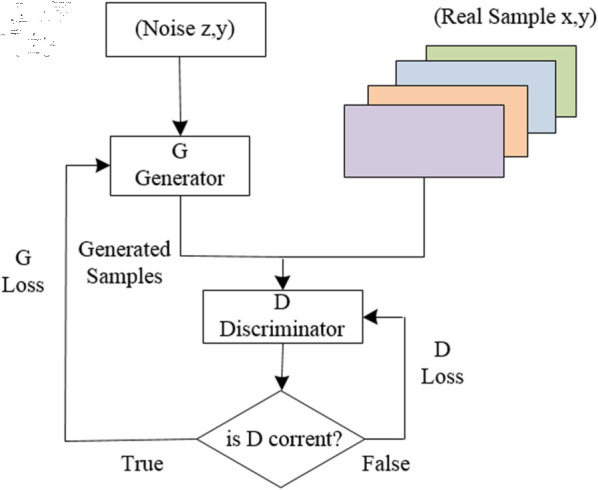
Diagram of GAN.

GAN is primarily composed of a Generator and a Discriminator, with the objective of producing sample data that closely resembles or is identical to real data, which can then be used for the training and analysis of various models. The fundamental principle and process of GAN generating samples are illustrated in Fig 14 [26]. The main approach involves adding noise to real samples to create approximate samples, which are then fed into the Discriminator along with the real samples for discrimination. Through multiple iterations, the GAN eventually generates approximate real samples that the Discriminator can no longer distinguish from the actual ones.

At present, a variety of different types of generative adversarial networks have been developed to suit various scenarios and conditions, including the traditional GAN, CGAN (Conditional GAN), and CWGAN (Conditional Wasserstein GAN), among others. Among these, CGAN [27] introduces constraint condition variables on the basis of GAN, enabling CGAN to generate sample data under conditional constraints, which has broader applications in image transformation and text-to-image synthesis. CWGAN [28], on the other hand, replaces the traditional cross-entropy loss function with the Wasserstein distance, further enhancing training stability and data quality. Building upon various GAN improvement schemes, this paper adopts the CWGAN-GP (CWGAN-Gradient Penalty) [29] approach to generate new APT malicious code samples. By using an improved loss function to generate label-specific samples, it can avoid mode collapse and training instability factors, and offers better adaptability to APT sample features that combine image and text data.

## 4 Experimental validation

### 4.1 Experimental environment setup

This experiment is built on a deep learning framework and the Python platform. The versions of the main systems and application platforms are listed in Table 3:

**Table 3:** Test platform parameters.

Hardware and software environment	Data Processing Platform	Virtual Machine Platform
Operating system	Windows 7	Windows 7
Software environment	anaconda /Python3.7	VMware15.1/python3.7
Memory	8G	4G
Display card	Nvidia GeForce rtx2070	-
Processor	Intel(R) Core(TM)i5	Intel VT-x

### 4.2 Evaluation metrics

To validate and compare the detection performance of the algorithms, this paper adopts publicly recognized and commonly used machine learning evaluation metrics. We first define the basic concepts:

- FP (False Positive): Samples that are actually negative but are predicted as positive.
- TN (True Negative): Samples that are actually negative and are predicted as negative.
- TP (True Positive): Samples that are actually positive and are predicted as positive.
- FN (False Negative): Samples that are actually positive but are predicted as negative.

These definitions are summarized in Table 4:

**Table 4:** Classification results matrix.

	Tested as positive sample	Predicted as Negative Sample
Actually negative sample	FP	TN
Actually positive sample	TP	FN

Therefore, the specific calculation formula is as follows [26]:

Pre(Precision):

$$Pre=\frac{TP}{TP+FP}\times 100\%$$ (4)

Re (Recall):

$$Re=\frac{TP}{TP+FN}\times 100\%$$ (5)

F1-score:represents the harmonic and average of accuracy and recall rates.

$$F1=\frac{2\times Re\times Pre}{Re+Pre}\times 100\%$$ (6)

Acc(Accuracy):

$$Acc=\frac{TN+TP}{TN+TP+FN+FP}\times 100\%$$ (7)

### 4.3 Experimental process and result analysis

To provide a comprehensive comparison and analysis of the experimental results, we first conducted experiments on the APT malware dataset using both single-feature and dual-feature methods. This allows for a complete comparison of performance differences among various machine learning algorithms. Through multiple experiments, the optimal parameters for the TCN algorithm used in this paper are shown in Table 5. The dilation rate refers to the spacing size in convolution operations. By using different dilation rates, the model can expand the receptive field size and learn more dependencies within sequences. The residual layers are set to 3 layers, the optimization function chosen is Adam, and the training runs for 100 epochs. A Dropout rate of 0.5 was set to avoid overfitting. The BiTCN and 1DCNN algorithm was configured with reference to the TN settings. The experimental data was divided into an 80% training set and a 20% validation set for classification and training.

**Table 5:** Parameter settings.

Parameter Name	Parameter Design
Expansion coefficient	(1,2,4)
Dropout	0.5
Epoch	100
Batch Size	50
Learning rate	Dynamic adjustment, min1e-6
Convolutional kernel	16×3
Residual layer number	3

#### 4.3.1 Single-feature experimental results and analysis.

Firstly, comparative experiments were conducted on the assembly instruction opcode features and binary image features of APT malware using different algorithms to analyze their recognition capabilities for different features.

1. Assembly Instruction Opcode N-gram Features

Following the principle outlined earlier, the N-gram features of opcode instructions extracted from malware samples of 12 APT groups were used for machine learning training, with N set to 3. To avoid excessive data volume and filter out invalid features, only N-gram features with a frequency greater than 500 were considered. It should be noted that reference [13] did not provide an experimental analysis process for the choice of the threshold 500. To save training time, this paper directly adopts this result for training. For N-gram features, the performance of conventional machine learning algorithms is shown in Table 6, with XGBoost, RF, and LR models demonstrating relatively better performance.

**Table 6:** Classification results of machine learning algorithms on N-gram features.

Model	Accuracy	Precision	Recall	F1-score
RF	0.9361	0.9376	0.9361	0.9355
Xgboost	0.9407	0.9404	0.9407	0.9399
DT	0.8885	0.8894	0.8885	0.8878
SVM	0.8896	0.8921	0.8896	0.8885
LR	0.9210	0.9215	0.9210	0.9206
KNN	0.8373	0.8368	0.8373	0.8351

**Fig 15:**
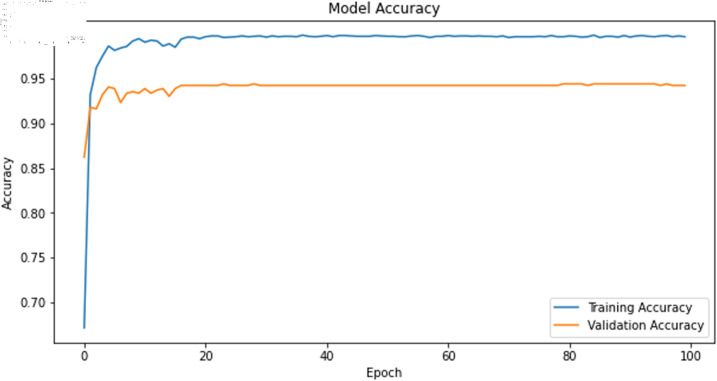
Training accuracy curve of TCN with N-gram features.

**Fig 16:**
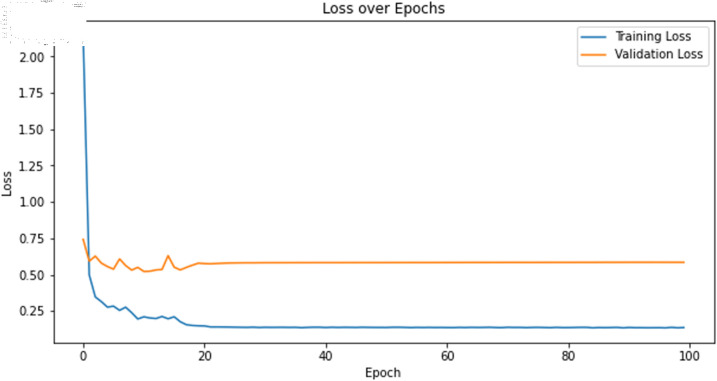
Training loss curve of TCN with N-gram features.

The primary deep learning training results are presented in Table 7, where the TCN, 1D-CNN, and BITCN models exhibit superior performance. The TCN model training loss and accuracy curves are illustrated in Figs 15 and 16, respectively.

**Table 7:** Classification results of deep learning algorithms on N-gram features.

Model	Accuracy	Precision	Recall	F1-score
1DCNN	0.9303	0.9304	0.9303	0.9299
LSTM	0.3298	0.2415	0.3298	0.2559
GRU	0.1415	0.0198	0.1415	0.0348
RNN	0.1405	0.0197	0.1405	0.0346
BiLSTM	0.2636	0.1672	0.2636	0.1574
TCN	0.9408	0.9431	0.9408	0.9414
BiTCN	0.9216	0.9228	0.9216	0.9213

2. Grayscale Image Features

To provide a comparison with the methods proposed later in this paper, for grayscale image features, we first convert the APT malware executable files into one-dimensional decimal grayscale image data based on the method described in reference [27]. It is important to note that since malware consists of continuous executable files, converting them into two-dimensional image data in a length-by-width format may disrupt their original data distribution patterns. Therefore, this paper directly converts them into one-dimensional sequences. After converting into one-dimensional decimal grayscale image data, due to the varying lengths of the samples, the data is truncated or padded to ensure uniform length for analysis. The training results of image-based machine learning algorithms are shown in Table 8. From the table, it can be observed that the XGBoost and RF algorithms exhibit relatively high recognition accuracy for image features.

**Table 8:** Classification results of machine learning algorithms with image features.

Model	Accuracy	Precision	Recall	F1-score
RF	0.8315	0.8432	0.8315	0.8296
Xgboost	0.8583	0.8602	0.8583	0.8560
DT	0.7804	0.7836	0.7804	0.7811
SVM	0.7502	0.7561	0.7502	0.7548
LR	0.7398	0.7449	0.7398	0.7390
KNN	0.7224	0.7268	0.7224	0.7136

**Fig 17:**
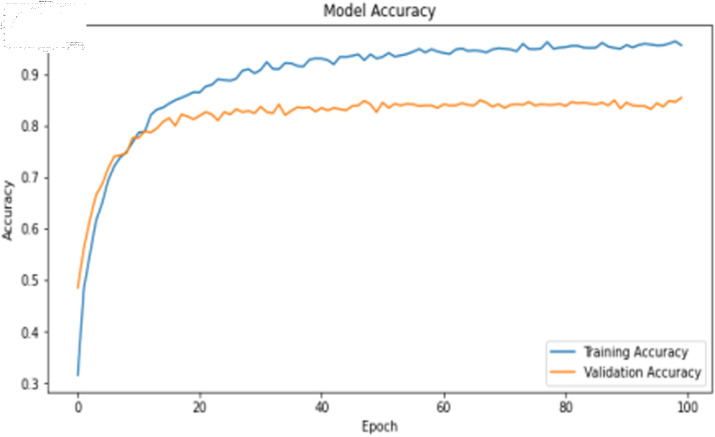
Training accuracy curve of 1D-CNN model with image features.

**Fig 18:**
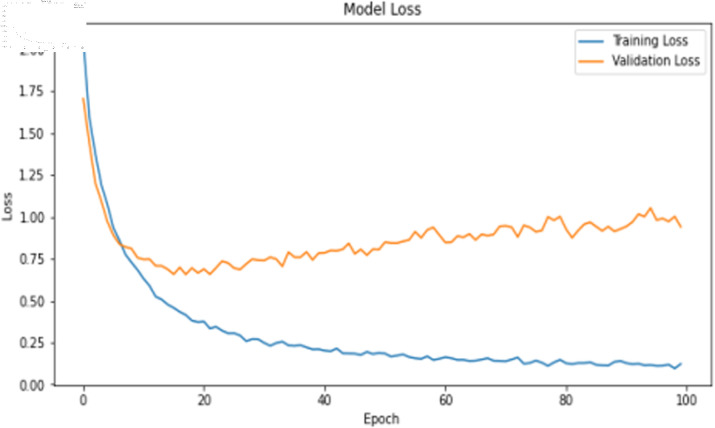
Training loss curve of 1D-CNN model with image features.

The deep learning training results are shown in Table 9. For image features, the 1D-CNN algorithm demonstrate relatively high training accuracy and precision, as illustrated in Figs 17 and 18. However, there is a noticeable gap between the training loss and validation loss for the 1D-CNN algorithms. This discrepancy may be attributed to imbalances or noise present in the original dataset.

**Table 9:** Classification results of deep learning algorithms with image features.

Model	Accuracy	Precision	Recall	F1-score
1DCNN	0.8548	0.8549	0.8548	0.8525
LSTM	0.3112	0.2444	0.3112	0.1939
GRU	0.4773	0.5704	0.4773	0.4338
RNN	0.1405	0.0198	0.1405	0.0347
BiLSTM	0.5098	0.5432	0.5098	0.4807
TCN	0.7793	0.8457	0.7386	0.7885
BiTCN	0.7770	0.7756	0.7770	0.7759

#### 4.3.2 Dual-feature experimental results and analysis.

The classification performance of various algorithms using single features has been analyzed above. Subsequently, the opcode N-gram features and grayscale image features are fused to form dual features for classification and recognition. Since the image data lengths of malware vary, the image data is first truncated or padded to a uniform length and then merged with the N-gram data to create a combined format of image data + N-gram data. Classification experiments are conducted based on the fused dual-feature data. The machine learning classification results are shown in Table 10. Comparing these results with those in Tables 6 and 8, it can be observed that the classification performance of XGBoost has significantly improved after feature fusion. However, the performance of other machine learning algorithms has not shown significant improvement, indicating that these algorithms did not effectively learn new information from the additional data.

**Table 10:** Classification results of machine learning with N-gram and image dual features.

Model	Accuracy	Precision	Recall	F1-score
RF	0.9233	0.9250	0.9233	0.9222
Xgboost	0.9465	0.9464	0.9465	0.9456
DT	0.8710	0.8731	0.8710	0.8686
SVM	0.8257	0.8295	0.8257	0.8256
LR	0.8188	0.8209	0.8188	0.8125
KNN	0.7328	0.7568	0.7328	0.7357

**Fig 19:**
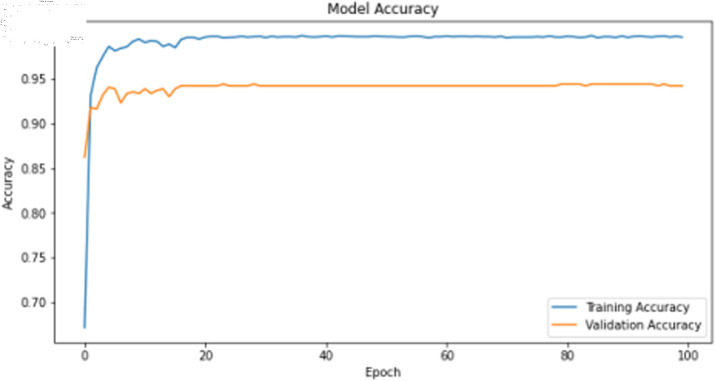
Training accuracy curve of TCN with N-gram and image features.

**Fig 20:**
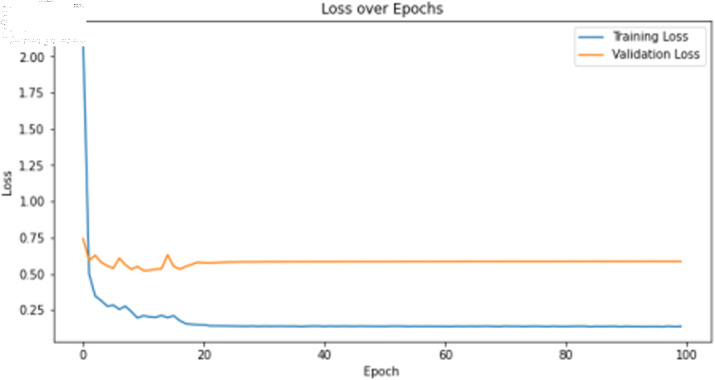
Training loss curve of TCN with N-gram and image features.

**Fig 21:**
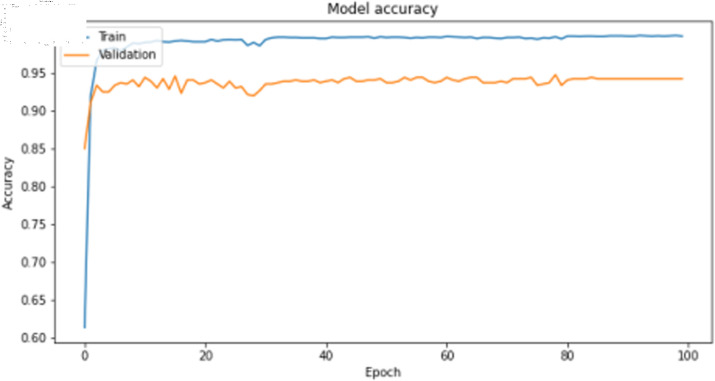
Training accuracy curve of BITCN with N-gram and image features.

**Fig 22:**
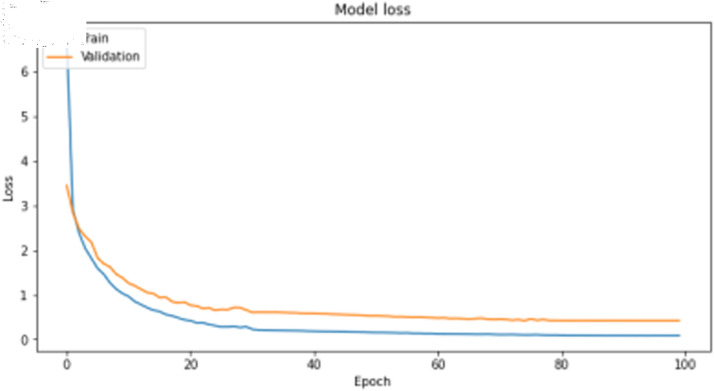
Training loss curve of BITCN with N-gram and image features.

The deep learning classification training results are shown in Table 11, where the training was conducted for 100 epochs. For dual features, both the TCN and BITCN algorithms demonstrate high training accuracy and precision, as illustrated in Figs 19, 20, 21, and 22. However, there is a noticeable gap between the training loss and validation loss for both TCN and BITCN algorithms, indicating a risk of overfitting.

**Table 11:** Classification results of deep learning with N-gram and image dual features.

Model	Accuracy	Precision	Recall	F1-score
1DCNN	0.9221	0.9240	0.9221	0.9224
RNN	0.2148	0.0905	0.2148	0.1218
GRU	0.1904	0.0903	0.1904	0.1012
LSTM	0.1405	0.0197	0.1405	0.0346
BiLSTM	0.1416	0.0200	0.1416	0.0350
TCN	0.9425	0.9590	0.9372	0.9480
BiTCN	0.9425	0.9418	0.9425	0.9416

#### 4.3.3 Experimental results and analysis of the new feature combination approach.

Subsequently, we proceed to validate and analyze the novel feature combination proposed in this paper, which employs the N-gram features combined with the frequency data of grayscale values from malicious code images. In this approach, the N-gram features remain unchanged, while the image features are modified from one-dimensional image data to the frequency data of 256-bit grayscale values. Subsequently, this data is concatenated and fused, then fed into various machine learning algorithms for validation and analysis,The results are shown in Table 12. It was observed that conventional machine learning algorithms achieved classification accuracy exceeding 99% with this new feature combination, with RF, SVM, and LR even reaching 100%.

**Table 12:** Comparison of machine learning algorithm results for N-gram + grayscale value frequency features.

Model	Accuracy	Precision	Recall	F1-score
DT	0.9972	0.9973	0.9972	0.9972
RF	1.0	1.0	1.0	1.0
SVM	1.0	1.0	1.0	1.0
LR	1.0	1.0	1.0	1.0
KNN	0.9954	0.9956	0.9954	0.9954
XGBoost	1.0	1.0	1.0	1.0

**Fig 23:**
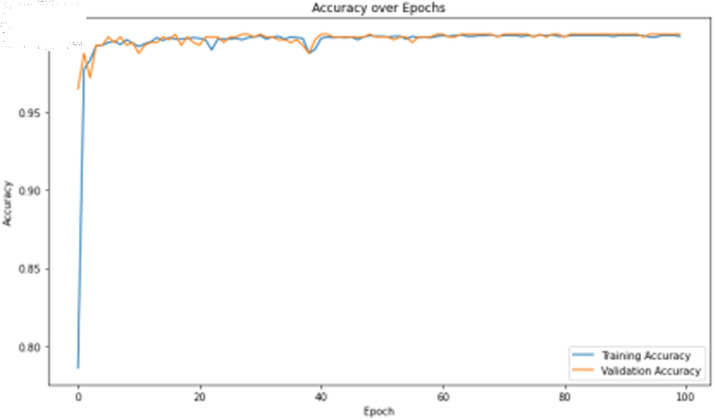
Training accuracy curve of TCN Model with N-gram + grayscale value frequency features.

**Fig 24:**
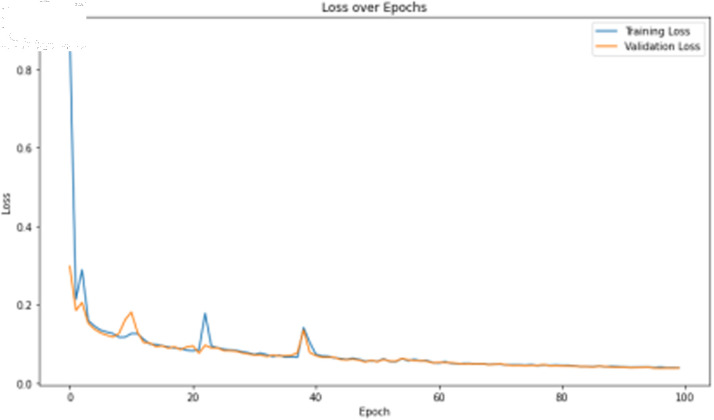
Training loss curve of TCN model with N-gram + grayscale value frequency features.

**Fig 25:**
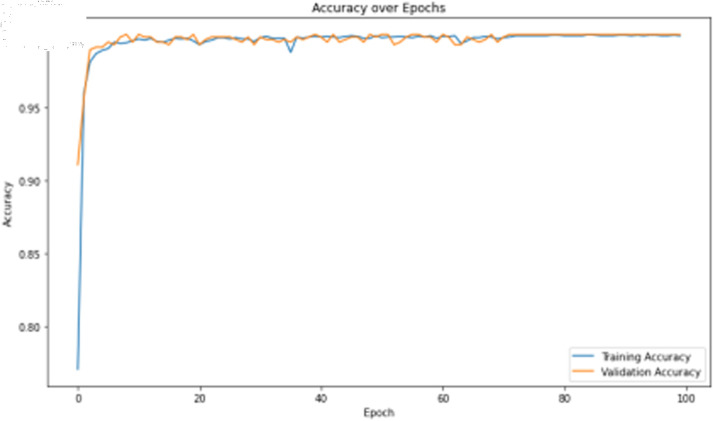
Training accuracy curve of BITCN model with N-gram + grayscale value frequency features.

**Fig 26:**
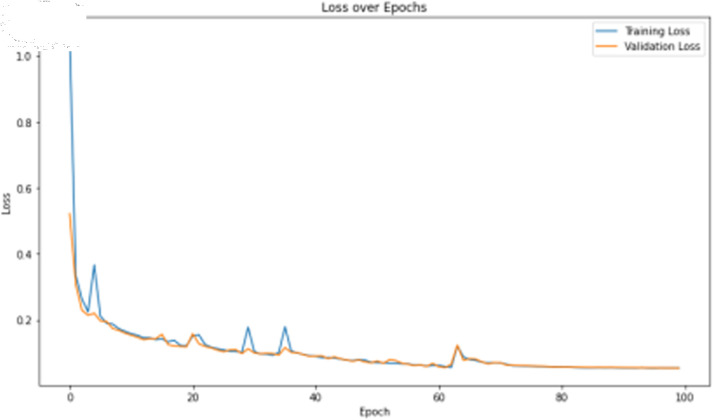
Training loss curve of BITCN model with N-gram + grayscale value frequency features.

**Fig 27:**
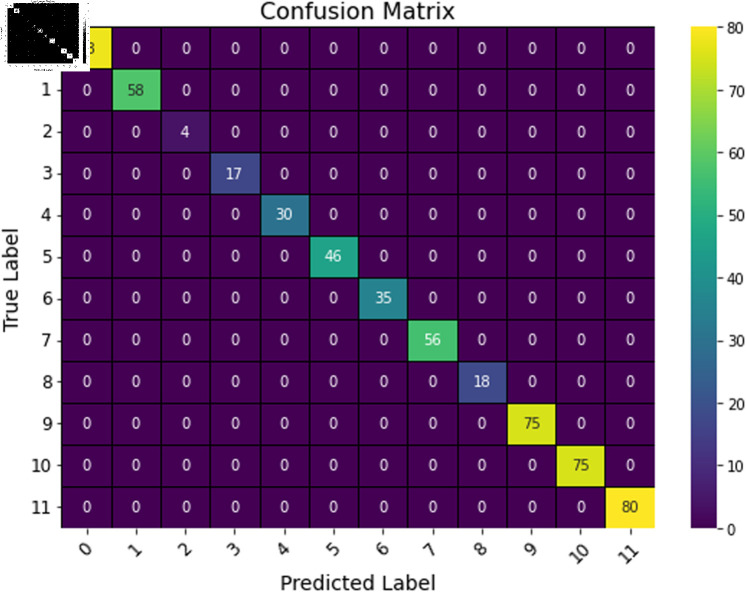
Confusion matrix of TCN model with N-gram + grayscale value frequency features.

The deep learning classification training results are presented in Table 13, where the training was conducted for 100 epochs. For dual features, both the TCN and BITCN algorithms exhibit exceptionally high training accuracy and precision, as illustrated in Figs 23, 24, 25, 26, and 27. The Figs demonstrate that the feature classification method proposed in this paper achieves excellent performance on both the training and validation sets, with no signs of overfitting. On the other hand, the performance of RNN, GRU, LSTM, and BiLSTM is suboptimal. Considering these results alongside previous findings, the overall performance of these algorithms is unsatisfactory, and they will not be included in subsequent training.

**Table 13:** Comparison of machine learning algorithm results for N-gram + grayscale value frequency features.

Model	Accuracy	Precision	Recall	F1-score
1DCNN	0.9965	0.9965	0.9965	0.9964
RNN	0.1625	0.0492	0.1625	0.0627
GRU	0.1678	0.0506	0.1678	0.0651
LSTM	0.1678	0.0506	0.1678	0.0651
BiLSTM	0.9283	0.9345	0.9283	0.9295
TCN	0.9998	0.9998	0.9998	0.9998
BiTCN	0.9982	0.9982	0.9982	0.9982

#### 4.3.4 Classification results and analysis after GAN augmentation.

**Fig 28:**
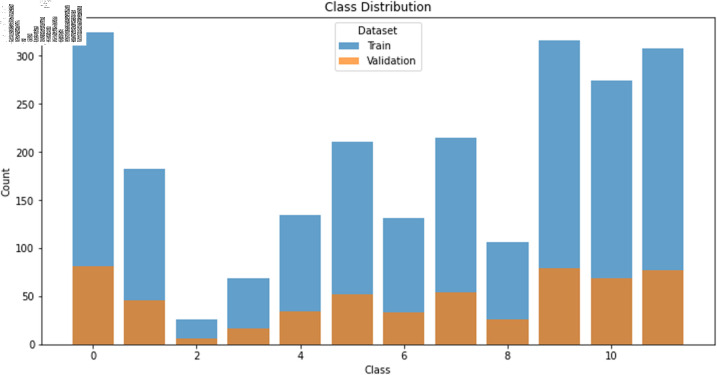
Data distribution results for 12 categories.

For the APT dataset D1, it is evident from Table 1 and Fig 28 that there is a significant issue of imbalanced distribution of sample quantities among the 12 organizations within dataset D1. APT 1 and Winnti organizations have a larger number of samples, whereas APT 19 and APT 21 have fewer samples, with APT 19 having only 32 samples. The sample quantities for other organizations also vary. The problem of uneven sample distribution and data imbalance within the dataset may lead to overfitting of the classification model and low classification accuracy. Therefore, to test the model’s effectiveness and mitigate the potential impact of data imbalance, this paper, after theoretical analysis and literature comparison, adopts the CWGAN-GP generative adversarial network method to increase the number of samples and balance the data distribution across different organizations.

In the process of utilizing CWGAN-GP to generate APT feature samples, after multiple rounds of experimentation, the model parameters were set as shown in Table 14, resulting in a smaller FID value, the highest quality of generated samples, and relatively stable training losses for both the discriminator and the generator. Here, G-lr represents the generator learning rate, which is the step size used when optimizing the generator, and D-lr is the discriminator learning rate, which is the step size used when optimizing the discriminator. An appropriate learning rate can accelerate the training process and help find the optimal solution for the model. Lambda_gp denotes the gradient penalty coefficient, where gradient penalty aids in maintaining the stability of the training process, prevents mode collapse, and promotes the generation of more diverse samples.

**Table 14:** Optimal stability parameters.

G_ lr	D_ lr	Lambda_ gp
0.00001	0.000001	15

In this paper, a data generation experiment was conducted on dataset D1 . Due to the limited number of original processed samples, totaling only 2,870 and exhibiting imbalance, the goal was to achieve a target data volume of approximately 10,000. To this end, the samples from all 12 organizations were uniformly expanded to 810 each, resulting in a total sample size of 9,720. After training with machine learning algorithms on the augmented dataset D1, the classification results are presented in Table 15. It can be observed that the primary machine learning models achieved classification accuracies exceeding 99.9%.

**Table 15:** Classification results of machine learning algorithms on dataset D1 after expansion

Model	Accuracy	Precision	Recall	F1-score
DT	0.9985	0.9985	0.9985	0.9985
RF	0.9995	0.9995	0.9995	0.9995
SVM	0.9990	0.9990	0.9990	0.9990
LR	0.9995	0.9995	0.9995	0.9995
KNN	1.0	1.0	1.0	1.0
XGboost	0.9990	0.9990	0.9990	0.9990

For the augmented dataset, the classification results after training with deep learning algorithm models are presented in Table 16. It can be observed that the 1DCNN, TCN, and BITCN algorithm models all achieved classification accuracies exceeding 99.8%.

**Table 16:** Classification results of deep learning algorithms on dataset D1 after expansion.

Model	Accuracy	Precision	Recall	F1-score
1DCNN	1.0	1.0	1.0	1.0
TCN	0.9980	0.9981	0.9980	0.9981
BiTCN	0.9990	0.9990	0.9990	0.9990

To further validate the applicability of the proposed method to smaller datasets of new APT organization samples, experiments were conducted on the APT organization sample dataset D2 constructed earlier, as shown in Table 2. This dataset contains only 274 samples, all of which are the latest APT samples. Due to the limited size of the sample dataset, direct classification training would lead to overfitting. Therefore, the dataset D2 was augmented using GAN, increasing the total number of samples to 10,000, with these samples evenly distributed across all organizations. The learning and training process was then continued, and the machine learning classification results are shown in Table 17.

**Table 17:** Classification results of machine learning on dataset D2 after expansion.

Model	Accuracy	Precision	Recall	F1-score
DT	0.9984	0.9985	0.9984	0.9984
RF	0.9994	0.9995	0.9994	0.9994
SVM	0.9984	0.9985	0.9984	0.9984
LR	0.9989	0.9989	0.9989	0.9989
KNN	0.9990	0.9990	0.9990	0.9990
XGBoost	0.9994	0.9995	0.9994	0.9994

**Fig 29:**
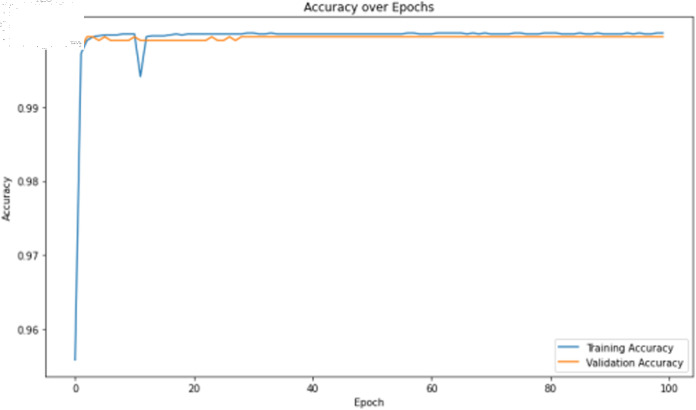
Training accuracy curve of the TCN model on dataset D2.

**Fig 30:**
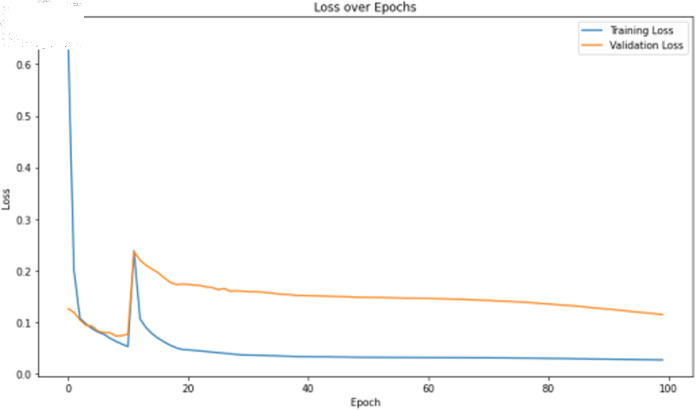
Training loss curve of the TCN model on dataset D2.

The classification results after training the self-constructed dataset D2 using deep learning algorithm models are shown in Table 18. As well as the training accuracy and loss curves of the TCN model are shown in Figs 29 and 30.

**Table 18:** Classification results of deep learning on dataset D2 after expansion.

Model	Accuracy	Precision	Recall	F1-score
1DCNN	0.9980	0.9981	0.9981	0.9980
TCN	0.9994	0.9995	0.9994	0.9994
BiTCN	0.9995	0.9995	0.9990	0.9997

From the above Tables 17 and 18 validation results, it can be concluded that both machine learning and deep learning algorithms achieve classification accuracies exceeding 99.8% after data generation and augmentation. In contrast, the classification accuracy without data augmentation drops to as low as 93.33%. It is evident that the data generated by GAN not only maintains but also enhances classification accuracy. Furthermore, this validates that the novel feature fusion method proposed in this paper remains precise and effective in identifying malicious code from new APT organizations.

Following this, we comprehensively evaluate and analyze the performance of the algorithms. We conducted three experiments on dataset D1, its augmented dataset, and the self-constructed dataset D2’s augmented dataset. Considering that the classification goal for APT malicious code detection typically aims to achieve a high detection rate while maintaining a low false positive rate, the F1 score is the most suitable metric for comprehensively evaluating algorithm performance, compared to accuracy and precision. The average F1 scores and detection times of the machine learning algorithms are shown in Table 19. From the table, it can be observed that the LR algorithm and the RF algorithm exhibit higher performance and lower detection times.

**Table 19:** Comprehensive performance comparison of machine learning algorithms for classification.

Model	Average F1-score	Detection Time (ms)
DT	0.9980	0.012
RF	0.9996	0.1
SVM	0.9991	0.1
LR	0.9995	0.03
KNN	0.9981	1.4
XGBoost	0.9995	0.1

We calculate the average F1 score, detection time, and parameter size of the deep learning algorithms, as shown in Table 20. From the table, it can be seen that the TCN algorithm maintains the best classification performance while keeping the detection time relatively low.

**Table 20:** Comprehensive performance comparison of deep learning algorithms for classification.

Model	Average F1-score	Detection Time (ms)	Parameter Size (M)
1DCNN	0.9982	1.9	3.14
TCN	0.9992	4.4	1.52
BiTCN	0.9988	9.4	3.04

Comparing the aforementioned machine learning and deep learning algorithms, it can be concluded that machine learning algorithms offer high detection accuracy and shorter processing times. However, it is important to note that traditional machine learning algorithms such as LR and RF are more suitable for handling relatively smaller datasets. Considering the multitude of APT organizations and the continuous growth and evolution of APT malicious code samples in the future, deep learning algorithms hold more advantages for tasks involving large datasets. Therefore, this paper recommends prioritizing the use of the TCN deep learning algorithm model for classification tasks, as it maintains high performance while consuming less time.

#### 4.3.5 Performance comparison with other models in literature.

The performance comparison is conducted with other models and methods under the same or similar APT dataset conditions, as shown in Table 21. From the table, it can be seen that the method proposed in this paper has reached the same number of samples as [30,31], with 3594 samples, and after GAN expansion, it has increased to almost 10,000, while the classification accuracy can still reach above 99.8%. Additionally, through validation with a self-constructed new APT sample dataset, it is proven that the method in this paper has better generalization capabilities. In terms of model parameter size and time efficiency, the listed literature does not provide specific model parameter sizes and running times, making direct efficiency comparison impossible. However, according to the research in [24], the TCN model’s efficiency is superior to major deep learning algorithms such as LSTM, GRU, and RNN. Therefore, the method in this paper maintains high accuracy while also having better operational efficiency.

**Table 21:** Comparison with similar research literature.

Literature	Number of APT samples	Features	Model	Accuracy
Han W *et al*. [19]	864	API	random forest	98.85%
A.D *et al*. [32]	190	Opcode	random forest	96.3%
Ishai R *et al*. [33]	1000	Raw Cuckoo reports	DNN	98.6%
Zhang J *et al*. [34]	2809	Opcode-Graph	GNNs	94.23%
Kida M *et al*. [30]	3594	Fuzzy Hashing	ML	89%
Wei C *et al*. [31]	3594	API	Dynamic LSTM	95.5%
Gil S *et al*. [35]	12665	API	LSTM	87.2%
This work	3594	Opcode + Grayscale Value	TCN	99.9%
This work	Expand to 9720	Opcode + Grayscale Value	TCN-GAN	99.8%

## 5 Conclusion

This paper innovatively proposes a method for tracing and classifying APT malicious code by combining the TCN algorithm with the GAN model. The method first innovates by extracting opcode and image grayscale value features from APT malicious code, then fuses the extracted features to generate hybrid features. Based on a publicly available APT attack dataset, the TCN algorithm achieves a classification accuracy of 99.5%. Considering the issue that the public APT dataset may have too few samples from individual organizations, potentially leading to data imbalance and affecting classification accuracy, the paper employs the CWGAN-GP generative adversarial network model to create a balanced and large-volume sample dataset. Experimental validation shows that the proposed method can still achieve a classification accuracy of over 99.8%. Finally, to verify the scalability and adaptability of the method, experiments conducted on a self-constructed small sample dataset using the TCN-GAN approach also yielded extremely high accuracy and low loss rates, indicating that the method retains the capability to classify and identify new and unknown APT malicious code. The research limitation is that it only considers static features for classification and tracing; the next step will be to investigate whether high-accuracy tracing and attribution can be achieved based on dynamic features. Additionally, the current collection of original sample data of malicious code from APT organizations is still limited, and further expansion of the sample dataset to include multiple different organizations is necessary in the future.
